# The crosstalk between m^6^A RNA methylation and other epigenetic regulators: a novel perspective in epigenetic remodeling

**DOI:** 10.7150/thno.54967

**Published:** 2021-03-04

**Authors:** Yanchun Zhao, Yunhao Chen, Mei Jin, Jin Wang

**Affiliations:** 1 Department of Pathology, Sir Run Run Shaw Hospital, School of Medicine, Zhejiang University, Hangzhou, 310000, China.; 2 Division of Hepatobiliary and Pancreatic Surgery, Department of Surgery, the First Affiliated Hospital, School of Medicine, Zhejiang University, Hangzhou, 310000, China.

**Keywords:** N^6^-methyladenosine (m^6^A), DNA methylation, chromatin remodeling, histone modification, non-coding RNA (ncRNA), RNA modification

## Abstract

Epigenetic regulation involves a range of sophisticated processes which contribute to heritable alterations in gene expression without altering DNA sequence. Regulatory events predominantly include DNA methylation, chromatin remodeling, histone modifications, non-coding RNAs (ncRNAs), and RNA modification. As the most prevalent RNA modification in eukaryotic cells, N^6^-methyladenosine (m^6^A) RNA methylation actively participates in the modulation of RNA metabolism. Notably, accumulating evidence has revealed complicated interrelations occurring between m^6^A and other well-known epigenetic modifications. Their crosstalk conspicuously triggers epigenetic remodeling, further yielding profound impacts on a variety of physiological and pathological processes, especially tumorigenesis. Herein, we provide an up-to-date review of this emerging hot area of biological research, summarizing the interplay between m^6^A RNA methylation and other epigenetic regulators, and highlighting their underlying functions in epigenetic reprogramming.

## Introduction

Epigenetics, which represents the modulation of heritable phenotypes without any alterations in DNA sequences, has become a significant regulatory mechanism of diverse physiological or pathological processes. The scope of epigenetics is extensive, typically including DNA methylation, chromatin remodeling, histone modification, non-coding RNAs (ncRNAs) and RNA modification 1. The first three members are superstars in epigenetics, and have been studied extensively so far. ncRNAs, mainly comprised of microRNAs (miRNAs), long non-coding RNAs (lncRNAs) and circular RNAs (circRNAs) 2, have provoked accumulating interests nowadays. In addition, there are more than 100 categories of RNA chemical modifications, and the common types include N^6^-methyladenosine (m^6^A), pseudouridine (ψ), 2'-O-methylation (Nm), m^1^A, 5-methylcytosine (m^5^C), adenosine-to-inosine (A-to-I), and N^6^, 2'-O-dimethyladenosine (m^6^Am) 3-7. Notably, m^6^A RNA methylation is the most abundant internal mRNA modification in mammals 8. With the rapid development of detection methodologies and high-throughput sequencing, the genome-wide features of m^6^A are being uncovered, which have increasingly attracted the attention of bioscience researchers.

**Figure 1:**
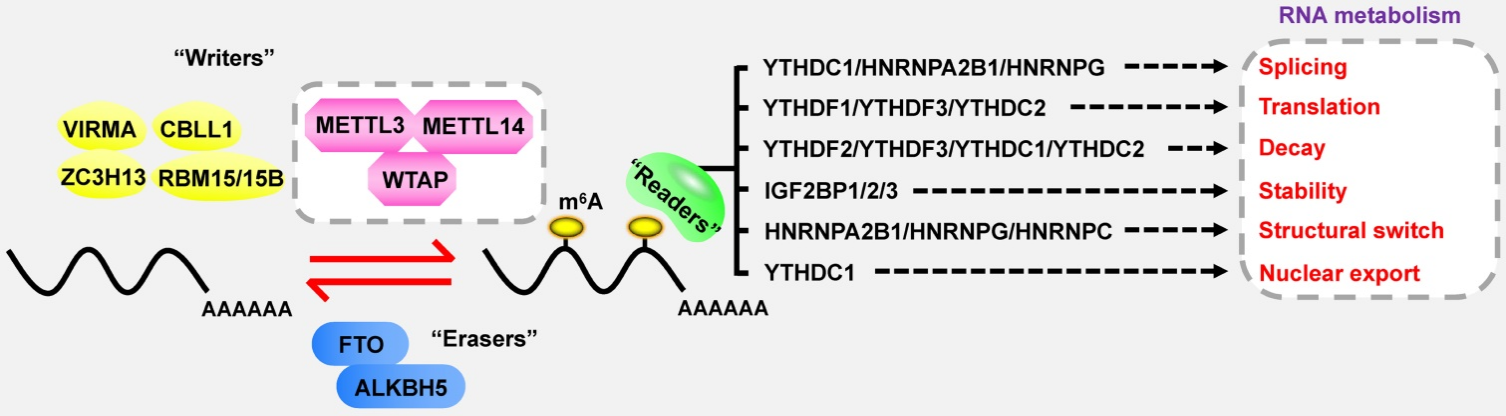
The dynamic and reversible processes of m^6^A modification. “Writers” deposit m^6^A methylation on RNAs, while “erasers” remove the m^6^A marks. Then “readers” are responsible for regulating the fate of targeted RNAs.

In the case of total RNA, m^6^A methylation occurs in approximately 0.1-0.4% of adenosines 9, predominantly located at 3' untranslated regions (3'UTRs), near stop codons and within the long internal exon 10, 11. DRACH sequences are verified as the consensus motif of m^6^A (D = G/A/U; R = G/A; H = U/A/C) 12. Strikingly, m^6^A modification is a reversible and dynamic process, which is deposited by methyltransferases (also called “writers”), and removed by demethylases (also called “erasers”) (Figure 1) 13, 14. Subsequently, m^6^A-binding proteins (also called “readers” or “effectors”) recognize and bind to the m^6^A marks of targeted RNAs to influence their RNA metabolism, including stability, translation, alternative splicing and transport 15-18. Furthermore, m^6^A plays a key role in far-ranging biological processes, such as cell differentiation, tissue development, environmental stress response, spermatogenesis, immune homeostasis and tumorigenesis 13.

Remarkably, it is commonly acknowledged that epigenetic regulations are intricate due to the interactions among epigenetic modifiers 19, 20. As a research frontier, m^6^A is just like a storm center to frequently interact with its peripheral partners, the other epigenetic modulators. These partners can be modified and regulated by m^6^A modification, while m^6^A methylation may also be efficiently controlled by these regulators 20-22. The coordinated relationships between m^6^A machinery and any other epigenetic counterparts elicit the epigenetic remodeling, which accounts for the perplexing modulations of various bioprocesses. Herein, we summarize the up-to-date findings about the interplay of m^6^A RNA methylation and other epigenetic modifications (Tables 1-3), and demonstrate how these associations impact biological functions, particularly in oncogenesis and tumor progression, highlighting the potential of m^6^A as a therapeutic target in the clinical practice.

## The genealogy of m^6^A modification

### m^6^A writers

The installation of m^6^A methylation is manipulated by the methyltransferase complex (MTC), which largely comprises of methyltransferase-like 3 (METTL3), methyltransferase-like 14 (METTL14), and Wilms tumor 1-associated protein (WTAP) 23. METTL3 functions as a key catalytic element to facilitate the formation of m^6^A, while METTL14 acts as an RNA-binding scaffold to promote the enzymatic activity of METTL3 24, 25. WTAP is responsible for the stabilization of the METTL3-METTL14 heterodimer and ensuring their accurate localization to nuclear speckles 26. Moreover, there are other co-factors involved in the conformation of MTC, including vir-like m^6^A methyltransferase associated (VIRMA, also known as KIAA1429) 27, Cbl proto-oncogene like 1 (CBLL1, also known as HAKAI), RNA-binding motif protein 15 (RBM15) with its paralogue RBM15B 28, and zinc finger CCCH domain-containing protein 13 (ZC3H13) 29, 30. Notably, METTL16 is another m^6^A methyltransferase, which dominates cellular SAM levels and mediates m^6^A modification of U6 small nuclear RNAs (snRNAs), pre-mRNAs or certain types of lncRNAs 31, 32. Additionally, METTL5 and ZCCHC4 have been identified as m^6^A methyltransferases for 18S rRNA and 28S rRNA, respectively 33, 34.

### m^6^A erasers

Fat mass and obesity-associated (FTO) and alkB homolog 5 (ALKBH5) are the only two known m^6^A demethylases to date. Although both demethylases belong to the AlkB family of dioxygenases, they eliminate m^6^A through different mechanisms. As the first identified m^6^A demethylase, FTO induces demethylation activity depending on the oxidative function, which requires iron (II) and α-KG 35. Specifically, FTO initially oxidizes m^6^A to form intermediate products, including N^6^-hydroxymethyladenosine (hm^6^A) and N^6^-formyladenosine (f^6^A), and subsequently hydrolyzes the products into adenosine, which is a sequential and multi-step procedure. However, the catalytic process mediated by ALKBH5 is a one-step reaction process, in which ALKBH5 directly abrogates m^6^A in an oxidative-dependent manner 36. Furthermore, a discrepancy has been observed in the recognition of substrates between ALKBH5 and FTO. ALKBH5 acts as an m^6^A-specific demethylase, while FTO can demethylate a variety of RNA modifications, such as m^6^A, m^6^Am and m^1^A 37.

### m^6^A readers

The m^6^A readers primarily consist of YT521-B homology (YTH) domain family proteins (YTHDF1/2/3), YTH domain containing proteins (YTHDC1/2) 15-17, 38, 39, insulin-like growth factor 2 mRNA-binding proteins (IGF2BP1/2/3) 40, and heterogeneous nuclear ribonucleoprotein (HNRNP) family (HNRNPA2B1, HNRNPC and HNRNPG) 41-43, which exert a great influence on the destiny of targeted RNAs.

As the first and most extensively investigated m^6^A reader, YTHDF2 can bind to the m^6^A residues in 3'UTR and facilitate RNA degradation 16, 44. Unlike YTHDF2, YTHDF1 selectively recognizes m^6^A marks in 5'UTR and near the stop codon, and boots the translation efficiency of targeted genes via the interaction with eukaryotic initiation factor 3 (eIF3) 15. Interestingly, YTHDF3 performs the dual functions of facilitating translation and inducing degradation of targeted transcripts 38. However, a recent research carried out by Zaccara et al. challenges the conventional views and reveals that there is no evidence to demonstrate the direct role of YTHDF proteins in promoting RNA translation 45. They also put forward an unified model of m^6^A function in which m^6^A modification predominantly affects mRNA degradation through the combined action of three redundant YTHDF proteins. These controversial viewpoints show the complex roles of YTHDFs in the m^6^A-based regulation, which require further discussion and verification. YTHDC1 is an m^6^A reader which not only regulates alternative splicing and nuclear export, but also accelerates mRNA degradation 17, 18, 22. YTHDC2 can induce the translation elongation of m^6^A-modified mRNAs, but also reduce the stability of certain targeted mRNAs 39, 46, 47. Furthermore, IGF2BPs are another cluster of readers whose K homology (KH) domains are required for m^6^A recognition. Generally, IGF2BPs can enhance the stability and translation of m^6^A-containing mRNAs 40, 48-52.

The binding of HNRNPA2B1 and m^6^A is mediated by a mechanism called “m^6^A switch”, in which alteration in the structure of targeted RNA caused by m^6^A methylation enhances the combination of m^6^A and HNRNPA2B1 41, 43. HNRNPA2B1 not only recognizes nuclear m^6^A-bearing transcripts to promote alternative splicing, but also strengthens primary miRNA processing 53. HNRNPG is capable of regulating alternative splicing or gene expression 42. Furthermore, HNRNPC may participate in the RNA processing of mRNAs or lncRNAs depending on m^6^A modification 41.

Strikingly, some reader-like effectors are also crucial for m^6^A regulation. For example, eIF3 can facilitate m^6^A-mediated translation 54. METTL3 has the capacity to promote translation of several mRNAs independent of its methyltransferase activity and other m^6^A-binding proteins 55. In addition, HuR is recognized to be involved in m^6^A-related events 56. However, the regulatory modes of HuR are currently controversial.

## The interplay between m^6^A and other epigenetic modifications

### m^6^A and DNA methylation

DNA methylation is a well-known and crucial epigenetic modification 57. Studies have revealed that DNA 5mC and 6mA methylations are the most common types of DNA modifications in eukaryotes and prokaryotes, respectively. Specifically, 5mC is generated by DNA methyltransferase 3A (DNMT3A) and DNMT3B 58, while demethylated either actively via ten-eleven translocation (TET) or passively by diluting DNA methylation labels during DNA replication 59, 60. In addition, N6AMT1 and ALKBH1 have been characterized as methyltransferase and demethylase of 6mA modification, respectively 61.

**Figure 2:**
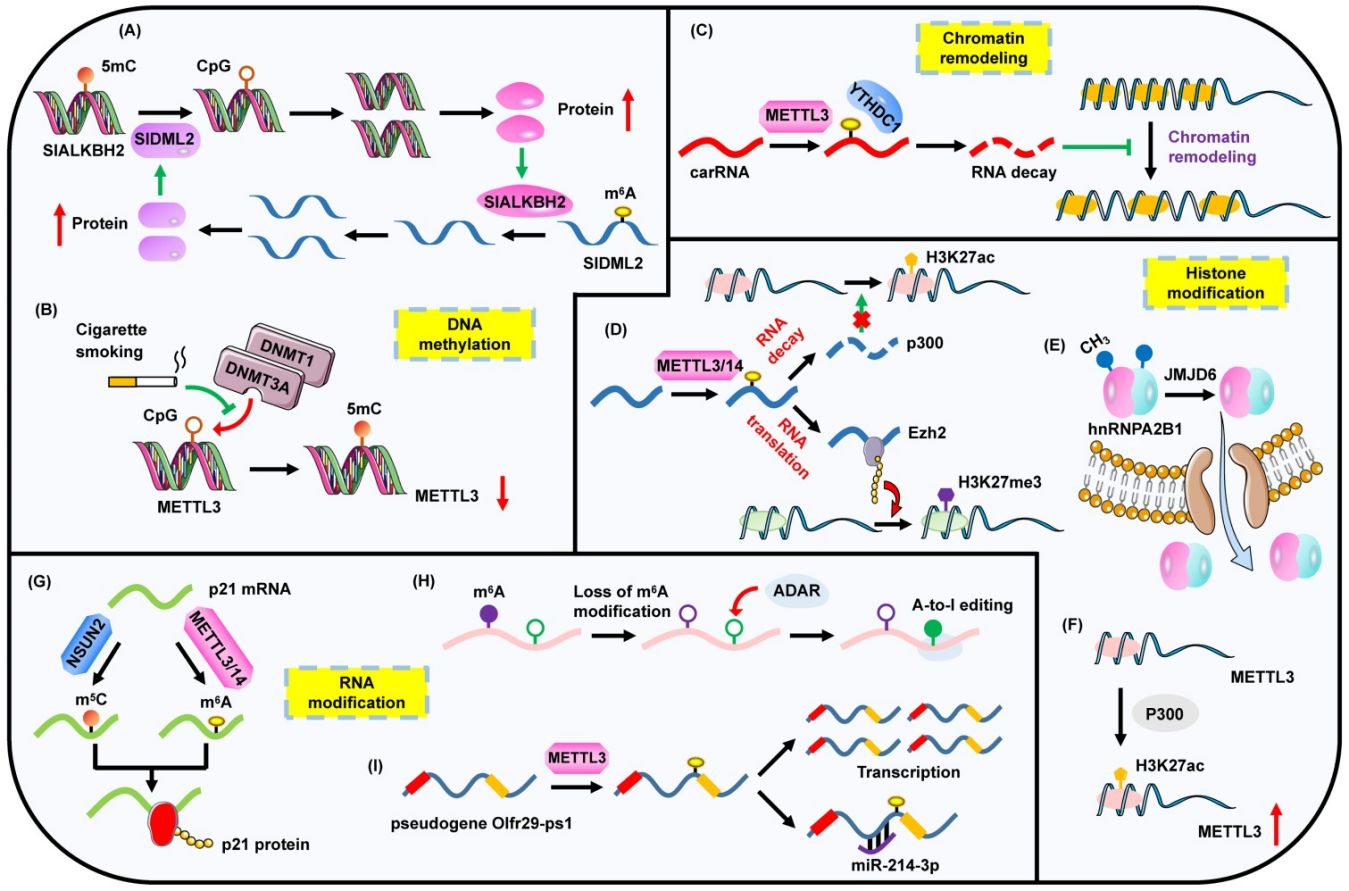
The complex interplay between m^6^A modification and other epigenetic regulators including DNA methylation, chromatin remodeling, histone modification and RNA modification. (A) The regulatory circuit of m^6^A modification and DNA methylation. (B) The loss of 5mC DNA methylation on METTL3 promotes its expression. (C) m^6^A methylation affects the chromatin state by regulating the expression of carRNAs. (D) m^6^A impacts histone modification through modulating the level of histone-associated enzymes. (E) JMJD6 mediates the demethylation of hnRNPA2B1, impelling its translocation to cytoplasm. (F) Histone acetylation facilitates METTL3 expression. (G) m^6^A methylation and m^5^C modification cooperatively promote the translation process. (H) Deficiency of m^6^A methylation leads to the enhanced level of A-to-I editing. (I) m^6^A methylation regulates the expression of pseudogene and impacts its sponge to miRNA.

Notably, an RNA methylome manifests the crosstalk between RNA and DNA methylation in fruit ripening 62. The m^6^A demethylase SlALKBH2, which is responsible for the decreased m^6^A levels of fruit-ripening genes, is modulated by SlDML2-contained DNA methylation. In turn, the stability of 5mC demethylase SlDML2 is strengthened by SlALKBH2-guided m^6^A demethylation (Figure 2A). Most recently, a comprehensive interplay between 5mC and m^6^A regulators across 33 cancer species based on bioinformatics analyses has been reported 63. The two types of methylations are functionally correlated with significant co-occurrences of genetic mutations. Some of the pivotal m^6^A/5mC genes are combined to establish an epigenetic module eigengene (EME). Interestingly, an elevated EME implies a strongly proliferative and aggressive cellular status, low inflammatory and immune infiltration, and enhanced enrichment of stromal signatures. Furthermore, EME level is useful to predict prognosis of cancer patients.

In pancreatic cancer, cigarette smoke condensate (CSC) is able to induce the hypomethylation of METTL3 through attenuating the bindings of DNMT1 and DNMT3a to the METTL3 promoter, which leads to the up-regulation of METTL3 and the following increased m^6^A levels of pri-miR-25 (Figure 2B) 64. However, CSC can also contribute to a diminished m^6^A abundance through triggering the hypomethylation of ALKBH5 CpG island in esophageal squamous cell carcinoma 65. Thus CSC seems to be a powerful factor to indirectly influence m^6^A modification via the straightforward impact on DNA methylation. Moreover, m^6^A profiling based on human fetal tissues reveals a preferential occupation of m^6^A on CpG-rich promoters. CpG-related promoters are capable of modulating m^6^A levels 66. These results may suggest the co-transcriptional process of m^6^A biogenesis and DNA methylation.

### m^6^A and chromatin remodeling

Chromatin remodeling is the rearrangement of chromatin state. An open or condensed state determines the accessible or unapproachable access for DNA binding proteins. Nowadays, several studies have demonstrated the crosstalk between chromain remodeling and m^6^A modification. BAF155 is a chromatin remodeling factor. RBM15 can negatively regulate the expression of BAF155 mRNA by decreasing its stability and promote its decay in an m^6^A-dependent manner 67. Notably, the regulation capacity of RBM15 on BAF155 requires the activity of METTL3. Furthermore, a reverse correlation between METTL3/YTHDC1 and chromatin accessibility in mouse embryonic stem cells (ESCs) is observed 22. Specifically, METTL3 promotes the m^6^A methylation of chromosome-associated regulatory RNAs (carRNAs), while YTHDC1 participates in the degradation of these m^6^A-marked RNAs. Thus METTL3/YTHDC1-guided m^6^A modification regulates the chromatin state and subsequent transcription by governing the expression of carRNAs (Figure 2C).

Remarkably, a study has revealed the deposition of m^6^A in chromatin-associated nascent pre-mRNAs from Hela cells. This m^6^A methylation, which is mainly present in exons and rarely in introns, is accomplished when mRNA is released into nucleoplasm. Surprisingly, m^6^A modification is required for the cytoplasmic mRNA stability of nascent transcripts, but not for the majority of splicing events 68.

### m^6^A and histone modification

Histone modification is a significant participant of post-translational regulations, which is involved in chromatin structure modulation, nucleosome dynamics and gene transcription. It primarily contains histone methylation, acetylation and ubiquitination. Interestingly, some modifications lead to the repression of transcription like H3K9me2/3 and H3K27me3, while others are associated with the activation of transcription including H3K4me1-3, H3K27me1, H3K36me1-3 and H3K27ac 69.

During cell development, METTL14 plays a vital role in the proliferation and differentiation of neural stem cells (NSCs). Surprisingly, the increased levels of H3K27ac, H3K4me3 and H3K27me3 modifications are observed when METTL14 is deleted. MTT assays demonstrate that m^6^A modulates the proliferation of NSCs partially via regulating H3K27ac and H3K27me3. Mechanistically, METTL14-mediated m^6^A methylation suppresses the stability of both CREB binding protein (CBP) and p300 transcripts which are the crucial modifiers of H3K27ac (Figure 2D) 21. In addition, METTL3-mediated m^6^A modification is necessary for neuronal development and neurogenesis. METTL3 regulates the m^6^A-modified histone methyltransferase Ezh2, which further advances the level of H3K27me3 (Figure 2D) 70. For erythropoiesis, m^6^A enzymes facilitate the translation of erythroid genes, especially those encoding SETD histone methyltransferases. The impairment of m^6^A leads to a substantial inhibition of H3K4me3 modification which is responsible for KLF1-centered transcriptional program required for erythropoiesis, heme synthesis or hemoglobin assembly 71. These studies suggest the divergent impacts on histone methylation induced by m^6^A, which may indicate that m^6^A-mediated histone regulation is cell-type-specific.

Apart from m^6^A-modulated histone modifications, histone modifiers also intimately participate in m^6^A rearrangement. The m^6^A reader hnRNPA2B1 is implicated in the immune response to DNA viruses. Herpes simplex virus-1 (HSV-1) infection induces the dimerization of hnRNPA2B1, which guides its nucleo-cytoplasmic translocation. Simultaneously, the arginine demethylase JMJD6 promotes the demethylation of hnRNPA2B1 at Arg226 and activates its translocation to cytoplasm, which further magnifies the expression of IFN-β (Figure 2E) 72. In gastric cancer, the promoter of METTL3 is marked by p300-regulated H3K27ac modification, which triggers the transcription of METTL3 and then leads to an elevated m^6^A level of HDGF (Figure 2F) 73. Furthermore, KDM5C-guided demethylation of H3K4me3 modification suppresses the transcription of METTL14 which can restrain the metastasis of colorectal cancer (CRC) via promoting the m^6^A level of SOX4 mRNA 74.

Moreover, two studies have afforded systematic evidence for the precise and dynamical deposition of m^6^A and histone modification. Huang et al. find that m^6^A peaks associated with H3K36me3 marks mainly locate near stop codons, while those H3K36me3 loci not modified by m^6^A are enriched in the coding sequence (CDS) or intron 75. The correlated positions imply their intertwined relationships. Intriguingly, although H3K36me3 cannot impact the expression of m^6^A key enzymes, it may affect the interaction between m^6^A enzymes and their targets. In other words, H3K36me3 is able to recruit m^6^A complex to deposit m^6^A imprinting. The fundamental element for the binding of m^6^A complex and H3K36me3 is METTL14 which is further identified to recognize H3K36me3 marks via a Pol II-independent pattern during transcription elongation 75. Additionally, Li et al. clarify that METTL3/METTL14-mediated m^6^A methylation modulates the levels of H3K9me2 76. The genome-wide correlation between m^6^A and KDM3B (H3K9me2 demethylase) is identified. To be specific, YTHDC1 recruits KDM3B to m^6^A-marked chromatin regions, triggering H3K9me2 demethylation and subsequent activation of gene expression. Conservatively, the co-occurrence of H3K36me2 and m^6^A is found in plants as well 77. All these investigations reveal the co-transcriptional interplay or even co-occupancy between m^6^A and histone modification.

### m^6^A and other RNA modifications

#### m^6^A and m^1^A

Currently, m^1^A is considered as a reversible modification in tRNAs, rRNAs, and mRNAs, which is methylated and demethylated by TRMTs and ALKBH1/3, respectively 78, 79. Remarkably, increasing evidence indicates a close link between m^1^A and m^6^A. Wei et al. discover that FTO has the ability to mediate both nuclear and cytoplasmic demethylation of m^1^A in tRNAs, and to subsequently suppress the RNA translation process 37. The special structure of FTO is analogous to the tRNA m^5^C methyltransferase NSUN6, which explains why another m^6^A demethylase ALKBH5 cannot recognize m^1^A at tRNAs as a substrate.

The m^6^A-binding proteins YTHDF1-3 and YTHDC1 are capable of directly binding to m^1^A sites. YTHDF2 accomplishes the recognition of m^6^A and m^1^A depending on its conserved residue Trp432 80. Functionally, YTHDF2 facilitates the degradation of m^1^A-modified transcripts 81.

Fortunately, two approaches including DART-seq and m^1^A-IP-seq/m^1^Aquant-seq, have been used to achieve genome-wide mapping of m^6^A and m^1^A with a single-base resolution, respectively 82, 83. However, further research should be conducted to explore the mechanisms between the two types of modifications via using the novel tools.

#### m^6^A and m^5^C

The m^5^C modification, which is the methylation of cytosine at carbon 5, is catalyzed by NSUN proteins and DNMT2 84, 85, and primarily occurs in tRNAs, rRNAs, and mRNAs 86. Previous studies have reported that m^5^C methylation is of great significance in the RNA stability, export and transcription 87.

Remarkably, there is a subtle relationship between m^5^C and m^6^A modifications. Courtney et al. demonstrate that murine leukemia virus (MLV) transcripts exhibit high levels of m^6^A and m^5^C modifications, which lead to a high level of viral replication. Mechanistically, the ectopic expression of YTHDF2 facilitates MLV replication, while the inhibition of m^5^C writer NSUN2 hinders MLV replication 88, which suggests that m^6^A may cooperate with m^5^C to engage in some biological events. Coincidently, a direct synergistic effect of m^6^A and m^5^C has been reported 89. METTL3/METTL14-catalyzed m^6^A methylation and NSUN2-induced m^5^C methylation can jointly enhance the expression of p21 mRNA in response to oxidative stress-triggered cellular senescence in tumor cells (Figure 2G). In addition to the cooperative relationship, an interaction between m^6^A and m^5^C has been observed. Specifically, METTL3/METTL14-mediated m^6^A modification can promote NSUN2-mediated m^5^C modification, and vice versa.

In addition, m^6^A reader YTHDF2 is capable of recognizing and binding to m^5^C in RNA 90. Deletion of YTHDF2 results in a remarkably expanded m^5^C level in rRNA. Interestingly, YTHDF2 participates in the regulation of pre-rRNA processing, which may be achieved via its modulation of m^5^C level.

#### m^6^A and A-to-I

The transition of A-to-I is processed by adenosine deaminases acting on RNA (ADAR) enzymes, which is a principal form of RNA editing 91. It is reported that A-to-I is a key factor influencing RNA metabolism, such as miRNA processing 92.

A reverse correlation between m^6^A and A-to-I has been identified using genomic analyses (Figure 2H). Loss of m^6^A modification contributes to the elevated level of A-to-I editing via a favorable association of ADAR with m^6^A-depleted transcripts. However, the underlying mechanism has not been fully elucidated. One possible reason for the occurrence is that the alteration of m^6^A-induced RNA structure may mediate the binding of ADAR and targeted genes. The occupation of m^6^A enzymes on RNAs may interfere with the localization of ADAR 4. However, whether A-to-I is capable of modulating m^6^A level remains indeterminate.

#### m^6^A and pseudogene

Pseudogene is a type of genomic element, which is partially homologous to corresponding functional genes, although lacks protein-coding capability due to mutations. Pseudogene widely participates in gene regulation 93.

Studies have revealed that there is a potential association between m^6^A and pseudogenes. *Olfr29-ps1* is a lncRNA pseudogene, which is stimulated by cytokine IL-6 in myeloid-derived suppressor cells (MDSCs). METTL3-mediated m^6^A methylation facilitates the expression of *Olfr29-ps1*, and simultaneously enhances its sponge to miR-214-3p (Figure 2I). Then MyD88, which is suppressed by miR-214-3p, is up-regulated to amplify the differentiation and immunosuppressive effects of MDSCs 94. Moreover, it is reported that m^6^A and pseudouridine (ψ) can collaboratively disrupt the binding of hPUM2 to its targeted RNAs 95.

In addition, m^6^A and ψ play a crucial role in immunity. Durbin et al. apply a well-accepted RIG-I-related platform to examine the immunosuppressive potential of various RNA modifications 96. The results reveal that either m^6^A or ψ negatively correlates with the alleviated innate immune signaling. Specifically, m^6^A-modified RNAs may poorly bind to RIG-I. Although ψ-containing RNAs can intimately interact with RIG-I, they are unable to initiate the canonical RIG-I antiviral signaling.

#### m^6^A and m^6^Am

When the transcription initiation nucleoside of mRNA is 2-O-methyladeonisine (Am), m^6^Am methyltransferase PCIF1 is capable of catalyzing methylation on its N^6^ position to further generate m^6^Am, which is dependent on the structure of 7-methylguanosine (m^7^G) cap 97-101. Studies have revealed that m^6^Am can reinforce the stability of transcripts 102, while the findings about its effects on translation are inconsistently identified. Akichika et al. illustrate that m^6^Am enhances the translation of capped mRNAs 97. However, another study suggests that m^6^Am may impede cap-dependent translation 98.

Several studies demonstrating the genome-wide landscape of m^6^A and m^6^Am have been conducted, which provide reliable evidence for their relationship 103-105. The conserved m^6^Am signals can be detected in WTAP and ALKBH5, while the non-conserved m^6^Am signals can be identified in METTL3. Additionally, the non-conserved m^6^A signals can be found in PCIF1 105. Furthermore, FTO has been demonstrated to target m^6^Am. Functionally, FTO is responsible for the demethylation of m^6^Am in snRNA 37. Nevertheless, additional functional relevance of m^6^A and m^6^Am remains to be explored.

### m^6^A and ncRNAs

m^6^A modification exists in almost all types of ncRNAs, especially in miRNAs, lncRNAs and circRNAs. They are all vigorous performers participating in extensive biological processes, particularly in tumor malignancy. The crosstalk of m^6^A and ncRNAs is pervasive and inspiring, extending the scope of epigenetics.

#### m^6^A-miRNA

miRNA is a short non-coding RNA (no more than 22 nucleotides), and links to a variety of biological processes such as tumor growth, drug resistance, cell differentiation, and cellular senescence 106. Initially, primary miRNA (pri-miRNA) is cleaved into precursor miRNA (pre-miRNA) by the microprocessor complex comprising of endonuclease Drosha and DGCR8 protein. After being transported to cytoplasm by exportin 5, pre-miRNA is further cleaved by Dicer to release the double-strands RNAs, which are then loaded onto an AGO protein constituting the RNA-induced silencing complex (RISC) 107.

**Figure 3:**
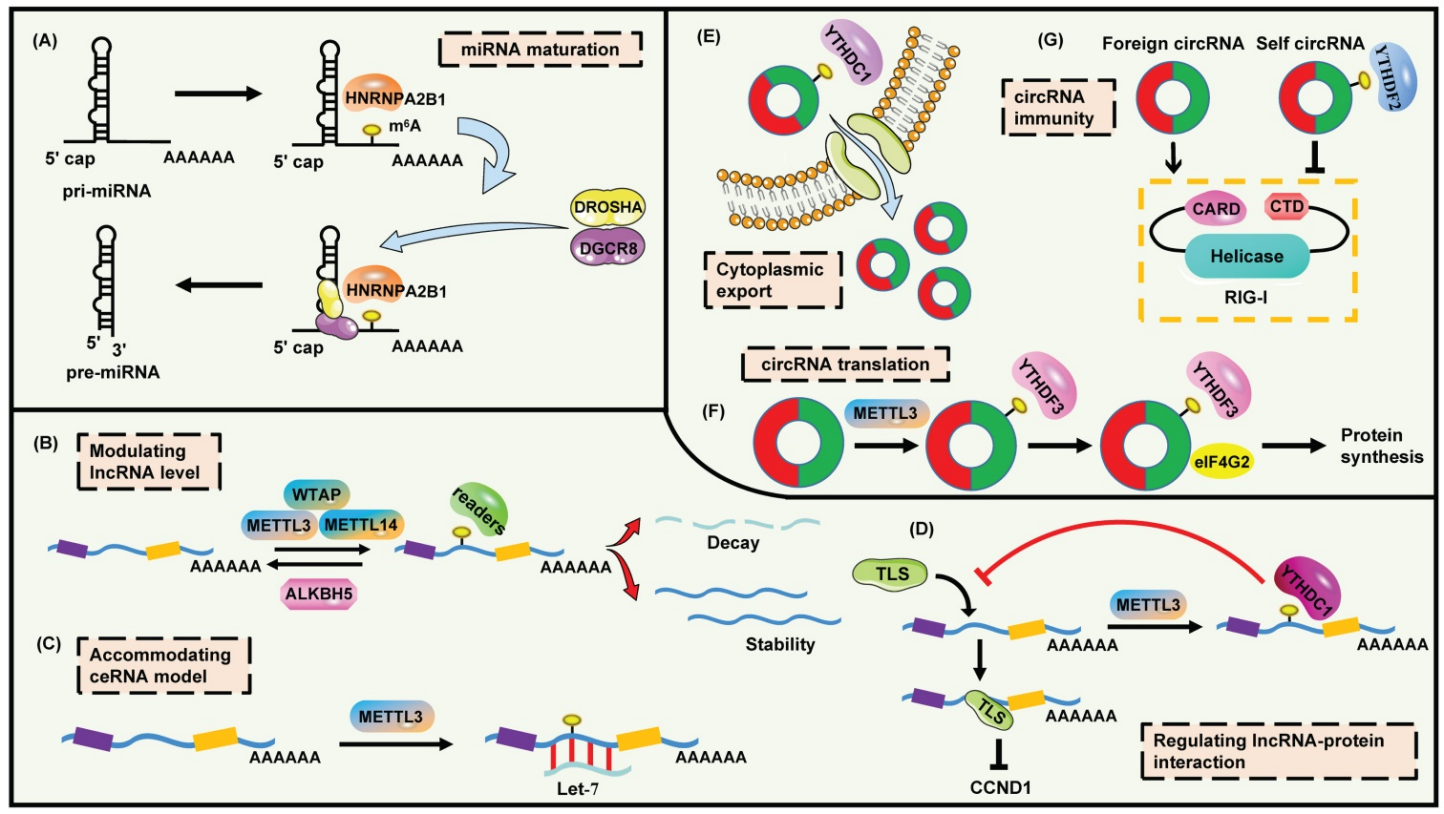
The functions and mechanisms of m^6^A modification on ncRNAs. (A) m^6^A promotes the maturation of miRNA. (B) m^6^A modulates lncRNA level. (C) m^6^A facilitates lncRNA to combine with miRNA. (D) m^6^A interferes the binding of lncRNA to proteins. (E) m^6^A mediates the cytoplasmic export of circRNA. (F) m^6^A regulates circRNA translation. (G) m^6^A assists the innate immune system to recognize self circRNA.

Intriguingly, m^6^A is the mark for advancing the processing of pri-miRNAs 108. METTL3 is sufficient to methylate massive pri-miRNAs to reinforce miRNA maturation through recruiting DGCR8 and m^6^A reader HNRNPA2B1 (Figure 3A). Moreover, HNRNPA2B1 interacts with DGCR8 to promote its binding to pri-miRNAs, which enhances the continuous generation of pri-miRNAs 53, 108. There are plenty of illustrations about this regulatory pattern. In bladder cancer, METTL3 accelerates cell proliferation by promoting the maturation of pri-miR221/222 which targets at PTEN 109. In CRC, METTL3 accounts for the aberrant m^6^A modification and boosts the production of mature miR-1246, which suppresses the SPRED/MAPK signaling 110. Wang et al. clarify that up-regulation of METTL3 blocks oxidative stress and apoptosis in colistin-evoked nephrotoxicity via the promotion of miR-873-5p mature process and the regulation of Keap1-Nrf2 pathway 111. Besides, mimicking the function of Dicer, METTL3-mediated m^6^A methylation leads to the splicing of pre-miR-143-3p which impairs VASH1 expression to facilitate angiogenesis and metastasis of lung cancers 112. Moreover, METTL3 increases the m^6^A modification of pre-miR-320 and drives osteogenic differentiation of bone marrow-derived mesenchymal stem cells 113. Another study demonstrates that the maturation of miR-7212-5p is impelled by METTL3-mediated m^6^A modification, while the miR-7212-5p/FGFR3 axis accounts for the regulation of osteoblast differentiation and fracture healing 114. In addition, an interesting study shows that CSC activates the excessive miR-25-3p maturation dependent on m^6^A mechanism in pancreatic cancer 64. The enhancement of METTL3 triggered by CSC contributes to the up-regulation of m^6^A level on pri-miR-25. Then NKAP serves as not only the m^6^A reader but also a splicing factor to stimulate the processing of pri-miR-25. Accumulating miR-25-3p suppresses PHLPP2, leading to the activation of AKT-p70S6K signaling 64. Except for METTL3, another m^6^A writer METTL14 also modulates the maturation of miRNAs analogously. As a suppressor in hepatocellular carcinoma (HCC), METTL14 interacts with DGCR8 to promote the processing of pri-miR-126 via an m^6^A-dependent pattern, triggering the enhanced level of miR-126 which represses the tumor metastasis 115.

Now that m^6^A is frequently enriched in 3' UTRs (near stop codons), and miRNA binding sites on mRNA are also commonly observed within 3' UTRs, the relationship between m^6^A and miRNA binding is discussed. However, an inverse localization pattern is identified 10. One reasonable explanation is that moderate spatial distance may be beneficial for mutual effects between m^6^A and miRNA. Actually, deficiency of m^6^A caused by loss of METTL3 or METTL14 restrains the miRNA-mRNA interaction as well as boosts HuR-mRNA interaction, which finally stabilizes the corresponding transcript 116. A more vivid example is provided by Zhang et al. 117. The m^6^A residue is found in the 3'UTR of YAP (353-357), and this modification is crucial for the conjugation of miR-582-3p and YAP. Hence, m^6^A modification may trigger the binding of miRNAs and targeted genes.

Additionally, AGO2 mRNA is highly methylated and positively modulated by m^6^A methyltransferases in human diploid fibroblasts. The miRNA abundance is controlled by m^6^A level based on the stability of AGO2 118. Knuckles et al. propose a model to delineate the RNA fate determined by m^6^A and microprocessor 119. In normal temperature, METTL3-centered complex deposits the m^6^A labels to massive RNAs containing mRNAs, pri-miRNAs, lncRNAs and snoRNAs, followed by the induction of their degradation mediated by DGCR8. However, acute heat stress leads to the re-localization of the m^6^A complex and DGCR8 at heat-shock genes to facilitate their decay. Meanwhile, those transcripts previously modulated by METTL3 and DGCR8 accumulate. This is an indirect fashion of m^6^A to control the degradation of miRNAs or other ncRNAs.

#### miRNA-m^6^A

A bidirectional relationship exists between miRNAs and m^6^A because miRNAs can regulate m^6^A-related events as well. Dicer, but not AGO protein, mediates the formation of m^6^A without altering the amount of methyltransferases or demethylases, and it may modulate nuclear speckle localization of METTL3 120. miRNAs are able to trigger de novo m^6^A methylation through a sequence pairing pattern. Moreover, miRNAs are responsible for the manipulation of the binding of METTL3 to miRNA site-containing mRNAs to affect m^6^A abundance, which is tightly associated with cell reprogramming to pluripotency 120. In HCC, miR-145 governs m^6^A level by inhibiting the expression of YTHDF2 121. METTL3 is targeted by miR-186, and it activates Wnt/β-catenin signaling in hepatoblastoma 122. miRNA let-7g which is inhibited by HBXIP, attenuates the expression of METTL3. Simultaneously, HBXIP is activated by METTL3 in an m^6^A-dependent manner 123. The positive feedback loop elaborates the complicated connection between miRNA and m^6^A.

#### m^6^A-lncRNA

LncRNAs are a group of non-coding transcripts longer than 200 nucleotides. The functions of lncRNAs are diverse, including regulating chromatin topology, serving as scaffolding for proteins or RNAs, governing RNA stabilization and transcription, or even producing peptides 124. LncRNAs can be modulated via multiple levels containing transcriptional regulation, post-transcriptional processing and degradation control 125. Importantly, the interaction between lncRNAs and m^6^A modification is a novel annotation (Figure 3B-D). Xiao et al. have generated the whole-transcriptome m^6^A landscape of human fetal tissues. Numerous lncRNAs are methylated by m^6^A especially in kidney, placenta and brain. Enhancer lncRNAs (originated from enhancers) have a higher enrichment in m^6^A modification compared with other lncRNAs. The distribution of m^6^A on lncRNAs is nearly balanced among 5'UTR, CDS and 3'UTR, which is different from the distribution on mRNA. Meanwhile, the proportion of m^6^A methylation on lncRNAs is lower than on mRNAs 66.

Metastasis-associated lung adenocarcinoma transcript 1 (MALAT1) is a highly conserved nuclear lncRNA which is closely related to the metastasis of tumors 126. As an abundant and essential transcript, MALAT1 is a paradigm to describe m^6^A-participated modification of lncRNAs. It is reported that m^6^A-modified MALAT1 (at A2577) is adequate for the binding of HNRNPC, one of the m^6^A readers which is essential for pre-mRNA processing 41. Similarly, m^6^A methylation (at A2515) increases the accessibility of MALAT1 for RNA-binding protein (RBP) through exposing its purine-rich sequences. Then HNRNPG, which governs gene expression and alternative splicing, binds to MALAT1 using its low-complexity region 42. In addition, the putative m^6^A writer METTL16 can interact with the U6 snRNA, pre-mRNAs and lncRNAs, such as MALAT1 31. To be specific, the 3' triple helix domain of MALAT1 is the binding site of METTL16 127. Jin et al. demonstrate that METTL3-guided m^6^A modulation contributes to the elevated expression of MALAT1 with the support of YTHDF3 in non-small cell lung cancer (NSCLC) 128. Then MALAT1 sponges miR-1914-3p to increase YAP activity and strengthen the metastatic potential of NSCLC.

XIST is another well-characterized mammalian lncRNA. It is the master regulator of X-chromosome inactivation (XCI), a dosage compensation process to balance X-linked gene expression via the suppression of transcription 129. Moindrot et al. employ a pooled shRNA screen to reveal that WTAP is one of key factors for XIST-mediated silencing, which co-localizes with XIST RNA in nuclear perichromatin spaces 130. Moreover, a following study demonstrates that XIST is heavily m^6^A methylated, and highlights the role of m^6^A modification in XIST-dependent transcriptional silencing 28. WTAP and METTL3 can be recruited by RBM15/15B to achieve the m^6^A modification on XIST. Then m^6^A-marked XIST is recognized by the reader YTHDC1, which promotes XIST-mediated gene inhibition 28. In addition, SPEN is a vital orchestrator of XCI by binding to XIST. The SPOC domain of SPEN is clarified to be involved in the recruitment of m^6^A machinery to XIST 131. Nevertheless, Nesterova et al. have conducted the systematic allelic analysis of XIST-mediated suppression in two interspecific mice models, and put forward the viewpoint that RBM15-centered m^6^A complex may provide minor contribution to this type of gene silencing 132. The possible causes for different consequences might rely on the redundancy of m^6^A modification and the various approaches to assay transcriptional inhibition. Although whether m^6^A can indeed control XIST-guided silencing is controversial, METTL14/YTHDF2 axis is feasible for regulating the stability of XIST 133. Therefore, the sophisticated m^6^A-XIST interaction deserves further explorations.

Actually, there are many other m^6^A-bearing lncRNAs which have been reported in multiple tumors with the major mechanisms of RNA stability regulation. The lncRNA FAM225A is overexpressed in nasopharyngeal carcinoma, and m^6^A modification is identified on FAM225A enhancing its RNA stability 134. In HCC, METTL3-mediated modulation contributes to the stabilization of LINC00958 which intensifies the HCC lipogenesis 135. Similarly, LNCAROD is up-regulated by METTL3/METTL14 in head and neck squamous cell carcinoma (HNSCC) and impels its tumorigenicity through preventing YBX1 from degradation 136. VIRMA induces aggressive phenotype of prostate cancer through sustaining m^6^A levels and abundance of the oncogenic lncRNAs CCAT1/2 137. DANCR, initially recognized as an anti-differentiation lncRNA, is strengthened by IGF2BP2 based on an m^6^A-modified site, and boosts stemness like properties of pancreatic cancer 52. ALKBH5-guided m^6^A demethylation enhances the expression of PVT1 with the assistance of YTHDF2 in osteosarcoma 138. In epithelial ovarian cancer, METTL3 increases the level of RHPN1-AS1 139. Besides, there are several studies about m^6^A-related lncRNAs in CRC. RP11 promotes the dissemination of CRC cells by regulating the epithelial mesenchymal transition (EMT). Specifically, the expression of RP11 is collaboratively regulated by METTL3 and ALKBH5. Elevated RP11 recruits the hnRNPA2B1 which recognizes and stabilizes the mRNAs of Siah1 and Fbxo45, thereby preventing the Siah1 and Fbxo45-dependent proteasomal degradation of Zeb1 140. Moreover, a study demonstrates a regulatory loop between lncRNA and m^6^A. LncRNA GAS5 combines with the WW domain of YAP and promotes YAP degradation via modulating its nucleo-cytoplasmic translocation. During CRC tumorigenesis, the m^6^A reader YTHDF3 interacts with m^6^A-modified GAS5 and promotes its decay, thus inhibiting the degradation of YAP. Accumulating YAP further activates the transcription of YTHDF3. This is a complicated but intriguing negative feedback loop between m^6^A and lncRNA 141.

Furthermore, other regulatory layers contain the m^6^A-mediated translation and the RBP-binding of lncRNA. *LINC00278* is an m^6^A-methylated transcript regulated by METTL3/METTL14/WTAP and ALKBH5. Studies have revealed that *LINC00278* encodes a tumor-suppressing micropeptide called YY1BM, which suppresses the combination of YY1 and androgen receptor, rendering ESCC cells more sensitive to nutrient deprivation. Mechanistically, m^6^A modification promotes the translation of YY1BM via a YTHDF1-dependent manner 65. *pncRNA-D* is an irradiation-triggered lncRNA which interacts with RBP TLS/FUS. The interaction of *pncRNA-D* with TLS is associated with CCND1 inhibition. METTL3 is responsible for the half-time of m^6^A-methylated *pncRNA-D*. YTHDC1 competitively inhibits the binding of *pncRNA-D* to TLS, thereby alleviating TLS-mediated suppression of CCND1. The decrease of m^6^A modification leads to a G0/G1 arrest in the cell cycle relying on CCND1 142.

In addition to tumorigenesis, m^6^A-lncRNA interaction is involved in other cellular procedures as well. Yang et al. find that linc1281 is indispensable for appropriate mouse ESC differentiation. The METTL3-dependent m^6^A mark in the last exon of linc1281 is responsible for not only its functional roles, but also the interactions with pluripotency-related miRNAs 143. For immune homeostasis regulation, m^6^A-modified lnc-Dpf3 controls the migration of dendritic cell (DC). It is well-accepted that although rapid DC migration is vital for initiation of immune defense, timely cessation of its trafficking is also indispensable for the avoidance of excessive inflammation. In the early stage, CCR7-mediated DC migration accelerates in response to CCL19/CCL21. However, during the late stage, CCR7 stimulation triggers the expression of lnc-Dpf3 by removing its m^6^A methylation and protecting it from YTHDF2-mediated degradation. Then lnc-Dpf3 binds to HIF-1α to suppress the HIF1α-dependent glycolysis and the migratory capacity of DC 144.

#### lncRNA-m^6^A

Apart from the m^6^A-lncRNA interaction, lncRNA is able to impact the m^6^A methylation as well. In CRC, LINRIS maintains the stability of the m^6^A reader IGF2BP2 through blocking its ubiquitination/autophagy-lysosome pathway, which facilitates MYC-mediated glycolysis 145. Moreover, the antisense lncRNA may reinforce the interaction of parent transcripts (mature or nascent) with m^6^A enzymes to control gene expression. For example, the up-regulation of ARHGAP5 is associated with chemoresistance in gastric cancer. In the nucleus, ARHGAP5-AS1 enhances the transcription of ARHGAP5 by binding to its promoter. Furthermore, ARHGAP5-AS1 can recruit METTL3 in the nucleus to induce the elevated m^6^A modification on ARHGAP5 mRNA, eventually facilitating the stability of ARHGAP5 146. Similarly, GAS5-AS1 enhances the stability of GAS5 by interacting with ALKBH5 which eliminates m^6^A modification in cervical cancer 147. In addition, FOXM1-AS increases the binding of ALKBH5 to FOXM1 pre-mRNA in glioblastoma. ALKBH5-triggered demethylation impels the effects of RNA-binding protein HuR, contributing to the elevated level of FOXM1 148. GATA3-AS promotes the interaction of KIAA1429 with GATA3 pre-mRNA in HCC 149. Recently, a study by Zhu et al. reveals another interesting regulatory mode. LncRNA LINC00266-1 can encode a small peptide which tightly interacts with IGF2BP1. The binding of peptide strengthens the recognition of IGF2BP1 on m^6^A-modified RNAs like c-Myc, further enhancing the stability of targets which are closely associated with CRC tumorigenesis 150.

#### m^6^A-circRNA

CircRNA is a species of covalently closed and evolutionally conservative circular transcript, mainly deriving from back-splicing of exons 151. The structure of circRNA is quite stable. It is broadly expressed in various kinds of specimens via a cell or tissue-specific manner. CircRNA is extensively involved in biological processes, such as developmental modulation, pathogenesis of heart diseases, chemoresistance and tumorigenesis 152. It primarily functions as the sponge of miRNAs (ceRNA), as well as participates in the interaction with protein, transcription, splicing regulation, and even the non-canonical translation 153.

The information of m^6^A-modified circRNAs is finite but attractive. Zhou et al. have established a genome-wide map of m^6^A-circRNAs in hESCs and Hela cells, and revealed the cell-type-specific patterns of m^6^A modification on circRNAs 153. There are several features about m^6^A-circRNAs. For example, circRNAs containing long single exons instead of multi-exons are more likely to be modified by m^6^A. m^6^A-circRNAs are commonly generated from those exons without m^6^A peaks in mRNAs. Like mRNAs, circRNAs are methylated by METTL3 and recognized by YTHDF1/YTHDF2 154. Park et al. prove that both linear and circular m^6^A-marked RNAs can be edited by the YTHDF2-HRSP12-RNase P/MRP axis 155. CircNSUN2 is an m^6^A-methylated circRNA which promotes the liver metastasis of CRC patients. The m^6^A motif “GAACU” on circNSUN2 is recognized by YTHDC1, which enhances circNSUN2 export from nucleus to cytoplasm (Figure 3E) 156.

It is inspiring to observe that circRNAs possess widespread m^6^A modification, which is adequate to drive protein synthesis with even a single m^6^A site. This cap-independent translation requires the assistance of eIF4G2 and YTHDF3 157, 158. As expected, the translation can be abolished by FTO, while enhanced by METTL3 or METTL14 157. Besides, circE7 is identified as an m^6^A-marked, cytoplasmatic and polysomes-associated circRNA. E7 oncoprotein is produced from the translation of circE7 human papillomavirus, while the mutation of possible m^6^A motifs strongly suppresses E7 protein expression 159. Timoteo et al. reveal that METTL3 regulates the m^6^A levels while YTHDC1 impacts the back-splicing of circRNAs. The cooperation of METTL3 and YTHDC1 regulates the biogenesis of various circRNAs including circ-ZNF609 which is translatable. Moreover, YTHDF3 and eIF4G2 recognize circ-ZNF609 to regulate its translation (Figure 3F) 158. Tang et al. identify that approximately half of spermiogenesis-related circRNAs are created via the back-splicing at m^6^A-enriched sites in linear mRNAs where start and stop codons are usually located. The outcome is that these circRNAs embrace m^6^A-accociated open reading frames (ORFs) in their junctions, which reveals the novel role of m^6^A in coding-circRNAs biogenesis 160. These results enrich the m^6^A-based non-canonical functions of circRNAs.

In addition, m^6^A-circRNAs are also involved in the immunoregulation and environmental stress response (Figure 3G). Foreign circRNAs, instead of self-counterparts, are efficient to trigger T cell activation and antitumor immunity *in vivo*. The m^6^A methylation patterns of exogenous and endogenous circRNAs are quite distinct. Mechanistically, unmodified foreign circRNAs heavily stimulate MAVS polymerization and interferon production after the RIG-I recognition. Nevertheless, m^6^A modification impairs activation of immune genes induced by endogenous circRNAs to prevent aberrant responses, which means that m^6^A can be the identity for self circRNAs. YTHDF2 is required for the suppression of circRNA-mediated innate immune signaling 161. Intriguingly, a transcriptome-wide profiling of m^6^A-circRNAs is revealed based on the hypoxia mediated pulmonary hypertension (HPH) model. The m^6^A abundance of circRNAs is diminished but its expression is increased in hypoxia. m^6^A-circRNAs are predominantly derived from encoding transcripts spanned single exons. Furthermore, the network of circRNA/miRNA/mRNA is also regulated by m^6^A in HPH. CircXpo6 and circTmtc3 are both m^6^A-modified and then down-regulated in HPH 162.

#### circRNA-m^6^A

However, studies about the functions of circRNAs on m^6^A modifications are rare. Recently, a study has revealed the role of circRNA-modulated m^6^A machinery in major depressive disorder (MDD) 163. CircSTAG1 is down-regulated in MDD animal models or patients with MDD. CircSTAG1 has the capacity to capture ALKBH5 to reduce its translocation into the nucleus. Then m^6^A modification is enhanced, which results in an increased degradation of fatty acid amide hydrolase (FAAH) mRNA and a subsequent decrease in depressive-like behaviors, as well as astrocyte loss. In short, circSTAG1 ameliorates MDD through inhibiting the translocation of ALKBH5 and then augmenting m^6^A levels of FAAH mRNA. Further researches should be conducted to elucidate the complex interactions between circRNA and m^6^A.

### The potential clinical values of m^6^A-centered epigenetic modifications

Nowadays, it is generally believed that epigenetic regulations exert a crucial role in the pathogenesis of various diseases. Therefore, exploring the possible pharmaceutical agents targeting epigenetic modifications seems to be a promising therapeutic strategy. For example, it is reported that DNA methyltransferase inhibitor (DNMTi), 5-Aza-2'-deoxycytidine, is able to enhance immunotherapy in esophageal carcinoma by promoting the expression of MAGE-A11 164. Moreover, histone deacetylase inhibitor (HDACi) MPT0B291 is capable of suppressing glioma growth partially via facilitating the acetylation of p53 165. Interestingly, the synergistic effects on treatment by combining multiple types of epigenetic inhibitors are widely reported 166-168.

In addition, inhibitors based on m^6^A-related enzymes have been actively investigated. However, current studies mostly focus on FTO, instead of m^6^A methyltransferases or m^6^A-binding proteins. As a highly selective inhibitor of FTO, meclofenamic acid 2 (MA2) can dramatically suppress the growth and self-renewal of GSC 169, 170. Chen et al. reveal that R-2HG can inhibit FTO and lead to the decreased stability of MYC and CEBPA, thereby impairing the proliferation of leukemia cells 171. There are other small molecule drugs targeting FTO that exerted substantial inhibitory effects in tumors, such as FTO-04 172 and FB23-2 173. Moreover, FTO inhibitors participate in the immunotherapy as well. In melanoma, FTO repression promotes tumor growth and increases the response of cancer to anti-PD-1 blockade 174. Analogously, the freshly recognized inhibitor of ALKBH5, ALK-04, is capable of reinforcing the efficacy of anti-PD-1 therapy 175. Furthermore, two series of adenine derivatives is identified as the selective inhibitors of METTL3, in spite of their elusive roles in clinical applications 176.

Notably, the intricate crosstalk between m^6^A and other epigenetic modifiers is tightly involved in tumor progression as mentioned above. Therefore, abolishing these interplay in human cancers may be the meaningful therapeutic perspective. For example, in gastric cancer, p300-guided H3K27ac modification can trigger the transcription of METTL3, eventually facilitating the malignancy of tumor 73. Perhaps, combination of HDACi and METTL3 inhibitors may become the feasible approach to interrupt the progression of gastric cancer. In addition, METTL3 mediates the m^6^A level of MALAT1 to increase its stability, which results in the drug resistance and metastasis of NSCLC 64. Possibly, suppressing the activity of METTL3 to regulate lncRNA levels may enhance the sensitive of NSCLC to cisplatin. Moreover, the diminished DNA methylation triggers the enhancement of METTL3, which further induces the maturation of pri-miR-25, promoting the development of pancreatic cancer 128. It is inspiring to try the combined treatment of DNMTi and METTL3 inhibitors to collapse the vicious axis of DNA methylation/m^6^A/ncRNA in this terrible cancer.

Nevertheless, all these ideas remain theoretical owing that investigations about drugs targeting at the crosstalk of m^6^A and other modifications are quite rare. The potential clinical values remind us that further explorations are urgently required.

## Conclusion

m^6^A RNA methylation, which is a new trajectory of epigenetic modification, has increasingly attracted the attention of researchers over the last few years. Studies have revealed that m^6^A plays a crucial role in RNA metabolism, such as degradation, alternative splicing, and translation. In addition, the interactions of m^6^A and targeted RNAs exert great influence on various biological processes, particularly in tumorigenesis. Meanwhile, accumulating evidence has deciphered the interplay between m^6^A and other epigenetic modulators (DNA methylation, chromatin remodeling, histone modification, RNA modification and ncRNAs), further unveiling the mysteries of epigenetic reprogramming.

Briefly, m^6^A and DNA methylation may exhibit a cooperative relationship, which relies on the interaction between m^6^A demethylase and DNA demethylase. In chromatin remodeling, m^6^A writers or readers are able to regulate the expression of chromatin-related RNAs, thus accommodating the chromatin state. However, there is still a dearth of information regarding the two crosstalk. For example, whether the regulatory loop between m^6^A and 6mA DNA methylation is available deserves further explorations. Notably, the complicated links between m^6^A and histone modification gradually emerge. m^6^A methylation modulates the status of histone methylation or acetylation, while histone modification also intends to affect the expression of m^6^A-related genes. The co-transcriptional regulation expounds the accurate deposition of m^6^A and histone modification, which determines the precise control of bioprocesses. Furthermore, m^6^A not only controls the level of other RNA modifications such as m^5^C and A-to-I editing, but also collaborates with them to govern multiple physiological processes. These shed light on the reciprocal associations of m^6^A and other RNA modifications and pave the way to further comprehend other types of RNA modifications. There is also a close relationship between m^6^A modification and ncRNAs, including miRNAs, lncRNAs and circRNAs. In most cancers, m^6^A machinery plays a promoting or suppressive role through altering the expression of targeted ncRNAs. In turn, ncRNAs regulate the stability and expression of m^6^A-assciated enzymes. It breaks the stereotype of ncRNAs and opens up a new paradigm for exploring the potential roles of ncRNAs. Nevertheless, the crosstalk between m^6^A methylation and circRNAs has not been clearly elucidated, particularly the function of circRNAs on m^6^A regulation.

Generally, interactions between m^6^A modification and other epigenetic members actively participate in the progression of tumors. These crosstalk can not only serve as the essential biomarkers for cancers, but also provide insightful mechanisms to develop the promising therapeutic strategies. Admittedly, these findings are only the tip of the iceberg. In the future, firstly, abundant efforts are still required to uncover more underlying roles of the interplay among these epigenetic modifiers and reach the deeper understanding of epigenetics in cancers. Secondly, it is imperative to explore potential remedies targeting at these interactions to reverse the erroneous epigenetic remodeling and reshape the balance. To be specific, perhaps the combination of m^6^A enzymes inhibitors and other modifiers inhibitors (DNMTi, HDACi, etc.) deserve validations in multiple tumors. It may be more attracting to directly target at the crosstalk instead of the modification itself. Moreover, it is noteworthy that the associations between FTO and other modifications are poorly investigated. FTO is the most unambiguous drug target with several selective inhibitors. Clarifying the mystery of crosstalk between FTO and other epigenetic members might guide to improve treatment efficiency of cancers.

## Figures and Tables

**Table 1:** The complicated interactions between m^6^A and other epigenetic modifications

Categories of epigenetics	Related components	m^6^A regulators	Mechanisms	References
DNA methylation	SlDML2	SlALKBH2	SlDML2-induced DNA methylation regulates the m^6^A demethylase SlALKBH2, while SlALKBH2-guided m^6^A demethylation strengthens the stability of 5mC demethylase SlDML2 in turn.	62
	DNMT1, DNMT3a	METTL3	The binding of DNMT1 and DNMT3a to METTL3 promoter is reduced by cigarette smoke condensate (CSC), leading to the hypomethylation of METTL3 and facilitating its expression.	64
	/	ALKBH5	The CpG island of ALKBH5 is hypomethylated by CSC, which increases ALKBH5 expression.	65
Chromatin remodeling	BAF155	RBM15	RBM15 accelerates the decay of chromatin remodeling factor BAF155 via the m^6^A methylation machinery.	67
	carRNAs	METTL3 YTHDC1	METTL3 promotes m^6^A methylation of chromosome-associated regulatory RNAs (carRNAs), while YTHDC1 mediates their degradation.	22
Histone modification	H3K27ac, H3K27me3, CBP, p300	METTL14	METTL14 not only alters H3K27me3 modification, but also regulates H3K27ac modification by destabilizing CBP and p300 mRNAs.	21
	H3K27me3, Ezh2	METTL3	METTL3 deposits m^6^A modification on histone methyltransferase Ezh2, which increases the level of H3K27me3.	70
	H3K4me3	METTL3, METTL14, WTAP	The m^6^A modification catalyzed by METTL3/METTL14/WTAP complex substantially strengthens H3K4me3 modification.	71
	JMJD6	hnRNPA2B1	Arginine demethylase JMJD6 activates hnRNPA2B1 through facilitating its demethylation at Arg^226^.	72
	H3K27ac	METTL3	H3K27ac modification on the promoter of METTL3 triggers its transcription.	73
	H3K4me3, KDM5C	METTL14	KDM5C-mediated demethylation of H3K4me3 suppresses METTL14 transcription.	74
	H3K36me3	METTL14	H3K36me3 mark recognized by METTL14 promotes the binding of m^6^A methyltransferase complex to adjacent RNA polymerase II, depositing m^6^A co-transcriptionally.	75
	H3K9me2, KDM3B	YTHDC1	YTHDC1 induces the H3K9me2 demethylation via recruiting KDM3B to the m^6^A-marked chromatin regions.	76
RNA modification	m^1^A	FTO	FTO mediates demethylation of m^1^A in tRNA.	37
		YTHDF1-3, YTHDC1	m^6^A-binding proteins YTHDF1-3 and YTHDC1 are capable of directly binding to the m^1^A sites.	80
		YTHDF2	YTHDF2 recognizes m^1^A-modified transcripts and mediates their decay.	81
	m^5^C	YTHDF2	m^6^A reader YTHDF2 and m^5^C writer NSUN2 cooperatively facilitate murine leukemia virus (MLV) replication.	88
		METTL3, METTL14	METTL3/METTL14-mediated m^6^A methylation and NSUN2-mediated m^5^C methylation collaborate with each other to strengthen the expression of p21 mRNA.	89
		YTHDF2	YTHDF2 binds to m^5^C in rRNA with the Trp432 residue, remarkably decreasing the m^5^C level.	90
	A-to-I	/	Loss of m^6^A modification contributes to the elevated level of A-to-I editing via the favorable association of ADAR with m^6^A-depleted transcripts.	4
	Pseudogene	METTL3	METTL3 elevates the expression of lncRNA pseudogene Olfr29-ps1 and facilitates its sponge to miR-214-3p.	94
		/	m^6^A modification and pseudouridine (Ψ) collaboratively weaken the binding of RBP hPUM2 to its targeted RNAs.	95
	m^6^Am	METTL3, WTAP, ALKBH5	The m^6^Am signal can be detected in METTL3, WTAP and ALKBH5, while the m^6^A signal is found in m^6^Am writer PCIF1.	105
		FTO	FTO is responsible for the demethylation of both m^6^A and m^6^Am modifications.	25, 37

**Table 2:** The specific molecular mechanisms and biological functions of m^6^A modification on ncRNAs

Categories	m^6^A-related enzymes	Non-coding RNAs	Mechanisms	Biological functions	References
m^6^A-miRNA	METTL3	miR-221/222	Promoting miR-221/222 maturation.	Accelerating cell proliferation of bladder cancer.	110
	METTL3	miR-1246	Facilitating miR-1246 maturation.	Promoting the metastasis of colorectal cancer.	111
	METTL3	miR-873-5p	Strengthening miR-873-5p maturation.	Blocking oxidative stress and apoptosis in colistin-evoked nephrotoxicity.	112
	METTL3	miR-143-3p	Enhancing miR-143-3p maturation.	Facilitating angiogenesis and brain metastasis of lung cancer.	113
	METTL3	miR-320	Increasing the m^6^A level of pre-miR-320.	Driving osteogenic differentiation of bone marrow-derived mesenchymal stem cells.	114
	METTL3	miR-7212-5p	Mediating miR-7212‐5p maturation.	Inhibiting osteoblast differentiation and fracture healing.	115
	METTL3, NKAP	miR-25-3p	Accelerating miR-25-3p maturation.	Promoting the progression of pancreatic cancer.	66
	METTL14	miR-126	Inducing miR-126 maturation.	Suppressing hepatocellular carcinoma metastasis.	117
m^6^A-lncRNA	METTL3, YTHDF3	MALAT1	Enhancing the stability of MALAT1.	Inducing drug resistance and metastasis of non-small cell lung cancer.	128
	WTAP	XIST	Co-localizing with XIST.	Participating in XIST-mediated silencing.	130
	METTL3, WTAP, RBM15/15B, YTHDC1	XIST	Promoting XIST-mediated transcriptional repression.	/	28
	METTL14, YTHDF2	XIST	Abolishing the stability of XIST.	Suppressing proliferation and metastasis of colorectal cancer.	133
	METTL3	LINC00958	Promoting the stability of LINC00958.	Increasing the lipogenesis of hepatocellular carcinoma.	135
	METTL3, METTL14	LNCAROD	Up-regulating the expression of LNCAROD.	Facilitating the progression of head and neck squamous cell carcinoma.	136
	VIRMA	CCA1/2	Increasing the m^6^A level of CCA1/2.	Inducing aggressive phenotype of prostate cancer.	137
	IGF2BP2	DANCR	Strengthening the stability of DANCR.	Enhancing stemness-like properties of pancreatic cancer.	52
	ALKBH5, YTHDF2	PVT1	Elevating PVT1 expression.	Promoting the tumorigenesis of osteosarcoma.	138
	METTL3	RHPN1-AS1	Enhancing RHPN1-AS1 expression.	Accelerating the proliferation and metastasis of epithelial ovarian cancer.	139
	METTL3, ALKBH5, hnRNPA2B1	RP11	Increasing the expression of RP11.	Triggering the metastasis of colorectal cancer.	140
	YTHDF3	GAS5	Promoting the decay of GAS5.	Inhibiting the tumorigenesis of colorectal cancer	141
	METTL3, METTL14, WTAP, ALKBH5, YTHDF1	*LINC00278*	Modulating the m^6^A modification of *LINC00278* and then affecting YY1BM translation.	Regulating the progression of cigarette smoking-related esophageal squamous cell carcinoma.	65
	METTL3, YTHDC1	*pncRNA-D*	Methylating *pncRNA-D* and inhibiting its binding to TLS.	Modulating cell cycle.	142
	METTL3	linc1281	Sustaining the interaction of linc1281 and pluripotency-related miRNAs.	Affecting the differentiation potential of embryonic stem cells.	143
	YTHDF2	lnc-Dpf3	Inducing the degradation of lnc-Dpf3.	Controlling the migration of dendritic cells.	144
m^6^A-circRNA	YTHDC1	circNSUN2	Recognizing circNSUN2 to enhance its cytoplasmic transport.	Facilitating colorectal carcinoma liver metastasis.	156
	METTL3, YTHDC1, YTHDF3	circ-ZNF609	Promoting circZNF609 translation.	/	158

**Table 3:** The underlying molecular mechanisms and biological functions of ncRNAs on m^6^A modification

Categories	Non-coding RNAs	m^6^A-related enzymes	Mechanisms	Biological functions	References
miRNA-m^6^A	miR-145	YTHDF2	Inhibiting the expression of YTHDF2.	/	121
	miR-186	METTL3	Suppressing METTL3 expression.	Inhibiting the growth and metastasis of hepatoblastoma	122
	let-7g	METTL3	Attenuating the expression of METTL3.	Accelerating the progression of breast cancer.	123
lncRNA-m^6^A	LINRIS	IGF2BP2	Maintaining the stability of IGF2BP2.	Promoting the aerobic glycolysis in colorectal cancer.	145
	ARHGAP5-AS1	METTL3	Recruiting METTL3 to methylate and stabilize ARHGAP5.	Strengthening the chemoresistance of gastric cancer.	146
	GAS5-AS1	ALKBH5	Interacting with ALKBH5 to demethylate and stabilize GAS5.	Suppressing the growth and metastasis of cervical cancer.	147
	FOXM1-AS	ALKBH5	Increasing the binding of ALKBH5 to FOXM1 pre-mRNA.	Facilitating the tumorigenicity of glioblastoma stem-like cells.	116
	GATA3-AS	KIAA1429	Enhancing the interaction between KIAA1429 and GATA3 pre-mRNA.	Accelerating hepatocellular carcinoma progression.	149
	LINC00266-1	IGF2BP1	Promoting the recognition of IGF2BP1 upon m^6^A-modified RNAs like c-Myc.	Strengthening tumorigenesis of colorectal cancer.	150
circRNA-m^6^A	circSTAG1	ALKBH5	Capturing ALKBH5 and reducing its intranuclear translocation.	Attenuating the depressive-like behaviors.	163

## Abbreviations

ncRNA: non-coding RNA
miRNA: microRNA
lncRNA: long non-coding RNA
circRNA: circular RNA
m^6^A: N^6^-methyladenosine
m^5^C: 5-methylcytosine
A-to-I: adenosine into inosine
m^6^Am: N^6^, 2'-O-dimethyladenosine
METTL3: methyltransferase-like 3
METTL14: methyltransferase-like 14
WTAP: Wilms tumor 1-associated protein
VIRMA: vir-like m^6^A methyltransferase associated
CBLL1: Cbl proto-oncogene like 1
RBM15: RNA-binding motif protein 15
ZC3H13: zinc finger CCCH domain-containing protein 13
FTO: fat mass and obesity-associated
ALKBH5: alkB homolog 5
YTH: YT521-B homology
YTHDFs: YTH domain family proteins
YTHDCs: YTH domain containing proteins
IGF2BPs: insulin-like growth factor 2 mRNA-binding proteins
HNRNPs: heterogeneous nuclear ribonucleoproteins
carRNA: chromosome-associated regulatory RNA
CRC: colorectal cancer
HCC: hepatocellular carcinoma
NSCLC: non-small cell lung cancer
